# Reduction of physiological stress by urban green space in a multisensory virtual experiment

**DOI:** 10.1038/s41598-019-46099-7

**Published:** 2019-07-12

**Authors:** Marcus Hedblom, Bengt Gunnarsson, Behzad Iravani, Igor Knez, Martin Schaefer, Pontus Thorsson, Johan N. Lundström

**Affiliations:** 1 Department of Forest Resource Management, Swedish University of Agricultural Sciences, Umeå, Sweden; 2 Department of Ecology, Swedish University of Agricultural Sciences, Uppsala, Sweden; 3 Department of Biological and Environmental Sciences, University of Gothenburg, Gothenburg, Sweden; 4 Department of Clinical Neuroscience, Karolinska Institute, Stockholm, Sweden; 5 Department of Occupational Health Sciences and Psychology, University of Gävle, Gävle, Sweden; 6 Division of Applied Acoustics, Chalmers University of Technology, Gothenburg, Sweden; 7 Monell Chemical Senses Centre, Philadelphia, Pennsylvania USA; 8 Department of Psychology, University of Pennsylvania, Philadelphia, USA; 9 Stockholm University Brain Imaging Centre, Stockholm University, Stockholm, Sweden

**Keywords:** Urban ecology, Olfactory system, Sensory processing, Human behaviour, Public health

## Abstract

Although stress is an increasing global health problem in cities, urban green spaces can provide health benefits. There is, however, a lack of understanding of the link between physiological mechanisms and qualities of urban green spaces. Here, we compare the effects of visual stimuli (360 degree virtual photos of an urban environment, forest, and park) to the effects of congruent olfactory stimuli (nature and city odours) and auditory stimuli (bird songs and noise) on physiological stress recovery. Participants (N = 154) were pseudo-randomised into participating in one of the three environments and subsequently exposed to stress (operationalised by skin conductance levels). The park and forest, but not the urban area, provided significant stress reduction. High pleasantness ratings of the environment were linked to low physiological stress responses for olfactory and to some extent for auditory, but not for visual stimuli. This result indicates that olfactory stimuli may be better at facilitating stress reduction than visual stimuli. Currently, urban planners prioritise visual stimuli when planning open green spaces, but urban planners should also consider multisensory qualities.

## Introduction

Air pollution, noise, and a lack of restorative environments are more profound in cities than in rural areas^1,2^, a condition that leads to stress symptoms in a significant portion of urban populations^3^. Because urbanisation is predicted to double over the next 30 years^4,5^, stress will most probably increase in city dwellers^6,7^. Urbanisation fragmentation and reduction of urban green spaces is problematic because green spaces reduce stress and increase well-being^8–11^. As the links between urban green spaces, stress, and spatial planning are complex, transdisciplinary studies are needed to create truly sustainable cities^12–14^. Although consensus exists about the positive health benefits of urban green spaces, more knowledge is needed about the mechanisms behind why and how green spaces reduce stress^11^.

Exposure to urban green spaces can generate cognitive, affective, and psychophysiological benefits that reduce stress and attention fatigue^15–17^. In 1989, Kaplan and Kaplan propose one of the more influential theories to explain the restorative effects of green space, Attention Restoration Theory^18^. This theory suggests that visiting natural environments such as urban green space and parks reduces stress by stimulating involuntary attention and thereby reducing directed attention. That is, natural environments seem to provide restful experiences, reducing the need for directed attention. However, Ulrich at al. have demonstrated that stressed individuals experience mental restoration in different types of natural environments but emphasise evolutionary theories as reasons for restoration rather than involuntary restoration^15,19,20^. Experiments testing Kaplan and Kaplan’s^18^ and Ulrich’s^19^ theories suggest that natural environments have relaxing effects on physiological responses such as lowering heart rate and lowering cortisol levels^15,21^, results that provide experimental support for the notion that exposure to natural environments induces relaxation.

Most studies, however, are based on subjective and indirect measures, such as questionnaires, the distance to urban green space from a house, and the amount of greenery linked to a neighbourhoods using Global Positioning Systems (GPS). These measures do not provide information concerning details of what is actually experienced or any direct physiological responses^9^. Understanding the details of what exactly enhances the experiences or physiological responses in natural settings is needed if we are to design and manage environments that encourage well-being. Some evidence suggest that natural areas provide higher well-being than parks^22^ as informal gardens are perceived as more restorative than formal gardens^23^. In addition, the biodiversity of species might affect perception of the environment^24–26^. Perception of urban environments is further influenced by age, environment-related attitude, gender, and place attachment^22,24,27,28^. More importantly, an often overlooked key factor is that surroundings are perceived with all sensory modalities, including visual (sight), auditory (sound), olfactory (smells), and tactile (touch), where the synergistic and additive effects are often greater than the sum of the individual inputs^29–31^. Although it is obvious that we live and act within a multisensory environment, the physiological importance of multisensory effects on human perception and perceived stress in urban green spaces is not well known.

Earlier studies emphasise the interdependence of visual and auditory stimuli as well as subjective well-being or subjective pleasantness rather than objective measures of stress reduction per se^32,33^. Generally, individuals respond negatively to the lack of non-visual natural stimuli while watching nature scenes, indicating that they missed ‘the smells and sounds’ of nature, describing the experience as ‘too quiet’^21,34^. Moreover, an earlier study combining visual and auditory features failed to address the importance, the potential synergy effects, and the potential domination of one stimulus over another^15^. Physiological stress recovery has been shown to be faster for natural sounds than for traffic noise. For example, Alvarsson *et al*. used an arithmetic task as a stressor and compared stress reduction using skin conductance level (SCL) and heart rate variability^35^. Their results suggest that natural sounds may provide additional restorative experience to the visual perceptions. Compared to visual and auditory stimuli, the impact of odours in urban areas has been scarcely studied and mostly by association studies (e.g., linking photos to smells and memory). Contrary to common beliefs, odours communicate a rich set of information^36^, and recent data demonstrate that the human sense of smell is better than most animals^37^. Some recent studies mapping ‘smellscapes’ in cities highlight their importance for people’s everyday lives^38^. Thus, combining visual with auditory and olfactory features should increase the validity of the perceived reality by creating a more realistic environment. In an experimental setting, details of the sensory features of an urban environment and the mechanistic responses, such as stress reduction, can be studied in isolation. It is therefore a key research topic to investigate how our senses jointly enhance the subjective experience of nature, self-evaluated perceptions, and associated physiological measurements^29^.

**Figure 1:**
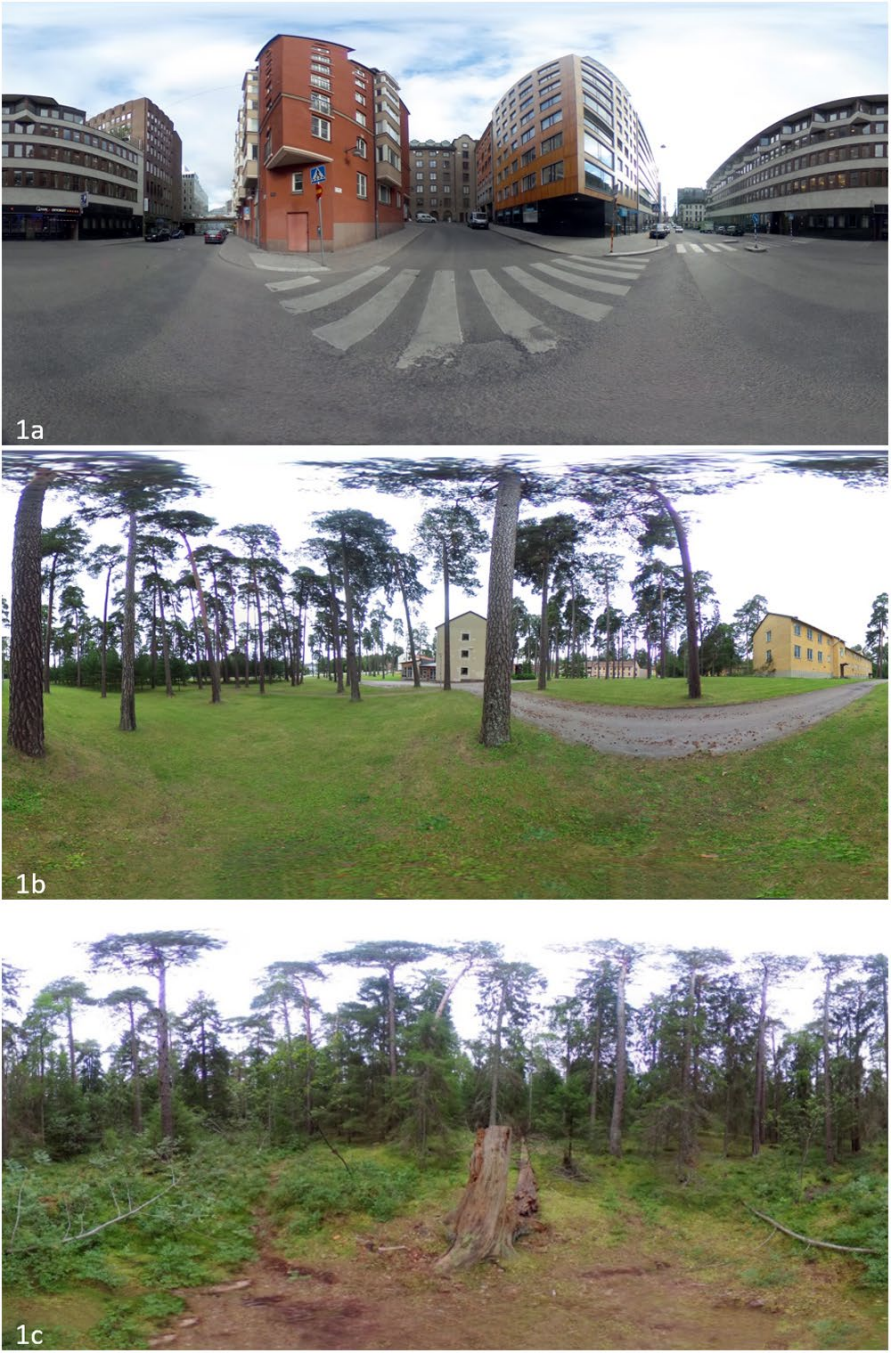
(**a**–**c**) Photos of the three environments, all located in Sweden: (**a**) urban area in Stockholm; (**b**) park in Uppsala; (**c**) city forest in Uppsala. The photos illustrate the Virtual 360 degree environments.

This study aims to determine the potential stress induction and stress recovery of three environments that, between them, display a step-wise reduction in green areas – an urban forest, a city park, and an urban environment (densely built up area) (Fig. 1). Stress is a multifactorial phenomenon that comprises both physiological and psychological components in a short- and long-term perspective. We focus on short-term stress and operationalise stress as skin-conductance level measurements^39^ given its reliance on autonomic responses and the clear link between the measure and physiological stressors. Each environment was represented by a multisensory combination of visual, auditory (e.g., slight breeze, traffic noise, and bird songs), and olfactory stimuli (e.g., urban smells such as diesel and natural smells of grass and fir). To facilitate a more ecologically valid representation of the environments^21,40,41^ while maintaining control of experimental stimuli, participants were exposed to these environments via 2D 360° Virtual Reality (VR) immersion in dynamic and synchronous stimuli.

Our specific hypotheses are that environments containing multisensory stimuli of green areas (forest and park) will attenuate physiological stress induction and facilitate a more rapid stress reduction compared to an environment void of green areas (i.e., urban areas). Merging senses, such as adding sound to a visual environment, will increase the overall positive (or negative) perception since, for example, bird songs have been previously shown to increase positive perception of urban photos^42^. In this experiment, we induce physiological stress using mild electric shocks to increase responses in the autonomic nervous system to the stressor for an increase in signal-to-noise ratio. We hypothesise that there is a positive relationship between the quality of green areas and the attenuation of stress induction as well as the speed of subsequent recovery from induced stress. Furthermore, we hypothesise that the forest environment, which naturally includes more diverse foliage, more bird species singing, and more smells, will reduce physiological stress more than the low diversity green space – i.e., the park environment. Our final hypothesis is that higher ratings of perceived pleasantness^32,33^ within the different senses (visual, olfactory, and auditory) are related to reduced physiological stress.

## Results

### Green environments lowered stress induction more than urban environments

Participants were exposed to one of three multisensory environments (a densely built up urban area, a park, or a forest) in which physiological stress was initially induced in a stress induction period (Stress period) containing five mild electric shock stimuli. The stress period was followed by a recovery period (Recovery period) to assess potential recovery in the absence of a stressor. Each environment was visually represented by a 2D 360° Virtual Reality photo. To each of these environments we added olfactory stimuli (city odours: diesel, tar, and gunpowder; park odours: grass; forest odours: two evergreen species and mushroom) and auditory stimuli (city noise: traffic; park noise: one bird; forest noise: nine bird species and sound of a slight breeze).

Mild electric shocks were used as a stressor during the Stress induction period. We determined whether electric shocks per se elevated participants’ physical arousal during exposure to the three environments by determining SCL increase within 6 s of the onset of the shock. Individual Student’s t-tests of baseline-adjusted skin conductance values (evoked responses) against the null value 0 indicated that physical arousal was significantly different from ‘no change’ in all environments (all t > 3.52, all *P* < 0.001), demonstrating significant responses to the stressor.

We then assessed whether there was a difference in skin conductance levels (SCL) between the Stress period and the Recovery period. To predict SCL, we assessed the coefficients of dependent variables, namely periods and environments, using Linear Mixed Models (LMM). Due to the multifactorial statistical model entered into the LMM, we subsequently statistically compared these individual coefficients with each other within each main fixed effect factor (Period and Environment) using separate marginal effects ANOVAs.

First, we found a main effect of Period for SCL performing marginal post-hoc ANOVAs on estimate coefficients (F(2,2471) = 26.71; *P* < 0.001) with an elevated trend across periods, from Baseline to Stress to Recovery. This technique demonstrated successful stress induction (Fig. S1). We then conducted a post-hoc t-test on the estimated coefficients of LMM. We found that Recovery had a higher stress level than Baseline, β = 0.95 (t(2471) = 6.758; *P* < 0.001), that Stress had a higher stress level than Baseline, β = 1.17, (t(2471) = 7.253; *P* < *0.001*), and that the Stress had a marginally higher stress level than the Recovery, β = 0.98, (t(2471) = 0.56; *P* > 0.28).

**Figure 2:**
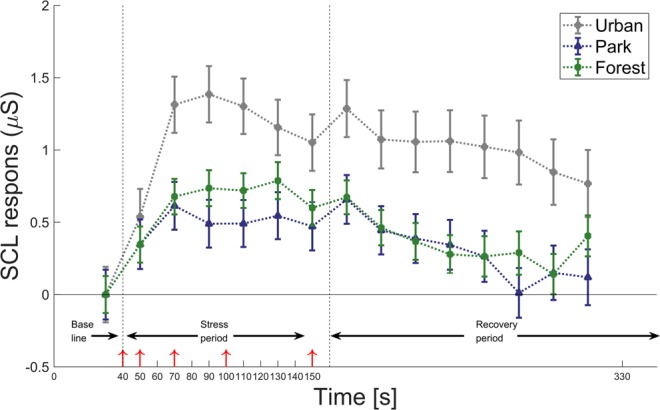
Average skin conductance levels (μSiemens) for the whole experiment linked to the Stress and Recovery periods for urban, park, and forest, including auditory and olfactory components. Red arrows indicate stress induction (electric shocks) at 40, 50, 70, 100, and 150 seconds. Error bars indicate Standard Error of the mean (SEM).

The three environments influenced SCL in different ways. As revealed by a LMM and following the same statistical path as described above, the effect of environment was F(2,2471) = 3.67 (*P* < 0.03). Based on the LMM, a post-hoc paired t-test on coefficients revealed that skin conductance levels during the whole experiment were significantly higher for the urban area than for the park, β = 2.02, (t(2471) = 2.45; *P* < 0.02). There was a similar trend between park and forest environments, β = 0.56, (t(2471) = 0.67, *P* = 0.25) (Fig. 2). Moreover, the average of SCL in all three environments were different for the Stress and Recovery periods (Table 1; average and standard deviation of recorded data). In other words, the respondents had high stress in the urban environment during both the Recovery and the Stress periods, whereas the respondents had significantly lower stress levels in the park during both the Recovery and the Stress periods.

**Table 1:** Means (M) of skin conductance levels (μSiemens) in the different environments. SD = standard deviation.

Stress			Recovery		
Urban	Park	Forest	Urban	Park	Forest
M = *1.139*	M = *0.4977*	M = *0.6532*	M = *1.0468*	M = 0.3323	M = 0.3538
SD = *4.0160*	SD = 2.902	SD = 2.3701	SD = 4.3801	SD = 3.0351	SD = 2.4693

### Effects between stress and recovery

**Figure 3:**
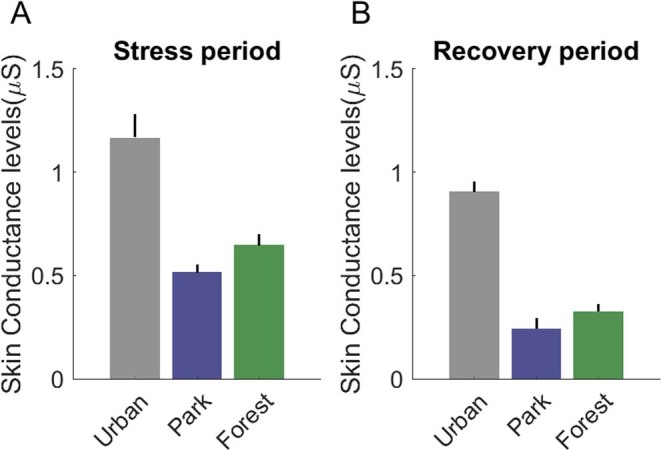
Baseline-adjusted skin conductance values (μSiemens), separated by environment and period. Errors bars indicate Standard Error of the mean (SEM). Marginal difference (*t* = 1.59, *P* < 0.15) in SCL between Urban A and Urban B, while Park A has a significantly higher SCL than Park B (*t* = 4.12, *P* < 0.002), and Forest A has a significantly higher SCL than Forest B (t = 4.98, *P* < 0.001).

During the three-minute recovery, participants exhibited a nominal but non-significant reduction in physiological stress levels when in the urban area. However, for both green areas physiological stress levels were significantly lower in the Recovery than in the Stress period (Fig. 3). Post-hoc tests of the coefficients of LMM revealed that skin conductance levels in the Stress period were higher for urban than for the park, β = 1.84, (t(2471) = 6.71; *P* < 0.001) and forest environments, β = 1.68, (t(2471) = 6.23; *P* < 0.001), but revealed no differences between the park and forest environment, β = 0.15, (t(2471) = 0.49; *P* = 0.31). In the recovery period, skin conductance was again higher in the urban environment relative to both the park, β = 1.60, (t(2471) = 6.59; *P* < 0.001) and the forest environments, β = 1.52, (t(2471) = 6.31; *P*  = 0.003), but no difference was found between the park and forest environments, β = 0.08, (t(2471) = 0.26; *P* = 0.39). In all three environments, there was no significant difference between the Stress and Recovery period (all t < 0.46 and all *P* = 0.32).

The LMM model contained by- participant random intercepts with standard deviation of 3.58 and range of [−9.39, 14.20]. Moreover, the model assessment has been performed by Akaike information criteria (AIC) that revealed model with interaction term was the best fit and had the lowest AIC = 7980 compared with the null model (AIC = 8071) and model with only fixed effect factor (AIC = 7985)^43^.

### Perceived pleasantness

The average perceived pleasantness (average of environments and sensory stimuli rated on a 1–100 scale, see Methods) was significantly higher for the park environment (M = 69.21, SD ± 11.1) relative to both the forest (M = 62.7, SD ± 12.74; *P* = 0.01) and urban area (*P* < 0.001), higher for the forest relative to the urban area (M = 37.19, SD ± 14.22; *P* < 0.001), and higher for the park relative to the urban area (*P* < 0.001). The ratings of perceived pleasantness differed significantly between the three environments across sensory stimuli (t = 90.01; *P* < 0.001; pairwise comparisons).

*Visual pleasantness* was significantly different for all environments but highest for the forest and lowest for the urban environments (see Table 2 for statistical values). *Olfactory pleasantness* was significantly different for all environments but highest for the park and lowest for the urban environment (Table 2). *Auditory pleasantness* was lowest for the city environment, and the auditory environment in the city was lower than the visual and olfactory ratings in the city. No significant differences were found between the sound in the forest and the park (Table 2).

**Table 2:** Pleasantness ratings of environments and sensory stimuli. M = average rating, SD = standard deviation, *P* = *significance*. Bold indicates highest rated environment in each sense. Pairwise Student’s t-tests used to assess differences.

Sensory	City	Park	*p*	City	Forest	p	Forest	Park	p
Visual	M = 54,00	M = 62.92	0.02	M = 54,00	**M = 74.15**	*p* < 0.01	**M = 74.15**	M = 62.92	0.003
	SD = 21.09	SD = 17.52		SD = 21.09	SD = 18.6		SD = 18.6	SD = 17.52	
Olfactory	M = 34.03	**M = 62.79**	*p* < .001	M = 34.03	M = 51.33	*p* = 0.01	M = 51.33	**M = 62.79**	0.004
	SD = 17.23	SD = 22.54		SD = 17.23	SD = 19.29		SD = 19.29	SD = 22.54	
Auditory	M = 29.88	M = 82.23	*p* < .001	M = 29.88	**M = 85.35**	*p* < 0.001	**M = 85.35**	M = 82.23	0.29
	SD = 21.2	SD = 9.98		SD = 21.2	SD = 11.67		SD = 11.67	SD = 9.98	

#### Relationship between perceived pleasantness and skin conductance

We found that odour (smell) was the only sense that predicted stress, where low perceived stress in the self-evaluations were linked to low stress responses in the physiological stress test (SCL –measures). To evaluate if the participants’ perceived pleasantness in an environment was important for the strength of the physiological stress response, we built a regression model where rated pleasantness of environments was entered as a predictor of each experimental period’s SCL. There was a significant relationship between perceived environmental pleasantness and SCL values in both the Stress (β = 1.44; r = 0.29; *P* = 0.004) and the Recovery period (β = 1.65; r = 0.29; *P* = 0.004). To further investigate the individual contribution of each environmental factor, we used individual step-wise regression models to assess how individuals’ rated pleasantness of each environment predicted their SCL response.

In the Stress period, only odour pleasantness significantly predicted SCL (β = −0.164; *P* = 0.04), whereas auditory pleasantness demonstrated a marginal significance (β = −0.18; *P* = 0.06), and visual pleasantness demonstrated a non-significant relationship (β = 0.013; *P* = 0.88). Similar results were demonstrated in the Recovery period, where only odour pleasantness significantly predicted SCL (β = −0.188; *P* = 0.03); auditory pleasantness demonstrated only a marginal significance (β = −0.45; *P* = 0.05), and visual pleasantness demonstrated a non-significant relationship (β = 0.013; *P* = 0.88).

### Relationship between subjective stress sensitivity and skin conductance

To evaluate whether participants’ self-rated *stress sensitivity* could be associated with the magnitude of the physiological stress response, individual values of participants’ Stress Sensitivity Scale were correlated with SCL during the Stress and Recovery periods. According to a bivariate Pearson correlation, there were no significant correlations between subjective stress sensitivity and SCL in either the Stress period (r = 0.05; *P* = 0.52) or in the Recovery period (r = −0.4; *P* = 0.66).

## Discussion

Here, we report on a virtual reality experiment in which the participants were first exposed to a physiological stressor and then recovered in one of three environments: a densely built up urban area, a park, or a forest. Unlike the urban environment, the green spaces reduced stress responses. We used a novel multisensory approach to add three senses simultaneously, a technique that provided a more realistic experience of the environment than previous experiments. Compared to sound and visual stimuli, odours had the largest effect on the stress response.

The results indicate that being in an urban area with no urban green space and with traffic noise and tar and diesel odours may keep an individual under sustained stress. Indeed, compared to exposure to urban environments, more natural environments such as parks and forests that include singing birds and natural smells lower stress levels within a minute of stressor offset. Our results confirm some of the sparse previous studies using an experimental environment that found physiological reduction of stress is associated with exposure to urban green spaces^15^. We can extend these findings to demonstrate that high perceived pleasantness ratings (psychological) of smells in park and forest environments were linked to lower stress responses (physiological) in a multisensory virtual reality environment. This finding was not demonstrated for visual stimuli, and our statistical tests demonstrate only a statistical tendency for auditory stimuli, which indicates that olfactory sensory inputs may be more important than visual and auditory features when creating environments that reduce stress.

### Stress and dose response

We found a link between physical stress reduction and the perceived environment. The densely built up urban environment had no vegetation and exposed the participants to traffic noise and diesel and tar smells. This environment did not reduce stress during the three minutes the test was conducted (only a nominal stress reduction) (Fig. 2), whereas the park and forest environment produced a significant reduction in stress response. Therefore, the results indicate that being in an urban environment is stressful, whereas being in a forest or park where birds are singing and the smell of grass and fir is prevalent produces a rather fast reduction in autonomic stress responses. However, exposure time in these environments is important. Previous studies showed that the rate at which people recover from stress differs with the experimental measure used; people experience the fastest stress recovery within the first 10 minutes when measuring heart rate and a slower recovery of 40 min when measuring cortisol levels^21^. Dolling *et al*. reported short-term effects of participants having ‘lesser’ stress symptoms when spending two hours in a forest^44^. However, whether time spent in urban green spaces increases well-being and reduces stress is still debated^45^.

Participants in this study experienced only a small nominal stress reduction in the urban environment after offset of the stressor. This finding was a somewhat surprising considering that the participants were recruited from Stockholm (about 1.5 million inhabitants) where they are presumably exposed to a higher degree of city stress in their daily lives and thus should be habituated to this type of exposure as being not too stressful. The majority of participants were young people; previous studies have found that older  participants compared to younger participants respond more negatively to noise^46^. Furthermore, we used electrical shocks as way to induce stress to enable fast stress responses for all participants. It is not known how the physiological stress response is specifically linked to psychological stress. The direct link between environmental stimuli and long-term stress cannot be directly assessed using an experimental design without undue burden on the research participants (is ethically difficult to conduct stress experiments on people already stressed). Future studies should assess whether age is a factor in the link between stress levels and exposure to green areas with the potential hypothesis that older people are less stressed by urban noise than younger people.

### Smell as a unique sense

The olfactory system has often been linked to stress-related aspects due to the uniqueness of its neural network. Whereas all other sensory systems connect to cerebral areas via a thalamic relay and connect to areas associated with stress processing via multi-synaptic pathways, the olfactory receptors are located only two synapses away from the amygdala and hypothalamus, the two key nodes in initial stress responses^47^. Moreover, olfactory stimuli benefit from unfiltered access to these glands due to the olfactory system’s non-obligatory thalamic coupling^48^. Combined with the seemingly automatic approach^49^ and avoidance^50^ to certain ecologically important odours, these unique anatomical features have promoted a link between odours and stress. Indeed, several previous studies have demonstrated links between either so-called ‘green odours’ and stress reduction^51^ or have determined that specific odours, such as lavender^52^, possess inherent stress-reducing properties. Unfortunately, most of these studies either lack control odours that are not stress-reducing or lack non-odour stimulus comparisons, which would correct for general pleasantness effects. In this study, even though the ratings of the odour stimuli were the least pleasant of the three sensory modalities, there was a clear link between odour pleasantness and stress reduction that was not present for the visual stimuli and not as clearly present for the auditory stimuli. Given the demonstrated link between auditory pleasantness and stress reduction, it is unlikely that the odours alone mediated the stress reduction effects. Nonetheless, our results indicate that odours associated with green areas with multisensory stimuli can contribute to stress reduction more so than odours associated with urban areas. However, these results need further study. We recently demonstrated that congruent odours significantly modulate how the brain processes multisensory visual and auditory stimuli. Specifically, we demonstrated that it is the later cognitive evaluation of the stimuli that is affected rather than the early perceptual response^53^. In other words, it is the interpretation rather than the perception of the stimuli that is affected. The exact mechanisms of the effects demonstrated in the results presented within this study are yet to be determined, but we argue that the potential multisensory effects on stress reduction may be caused by associations between the sensory stimuli and past experiences rather than by innately-derived mechanisms.

### Multisensory perceptions

Our results suggest that multiple senses can be used in experiments to evaluate respondent’s perceptions and that some senses may have stronger effects than other senses such as odour. It is difficult to statistically separate the importance of each of the three senses to the physiological response because we did not perform all the needed stimulus combination iterations. Both Annerstedt *et al*.^21^ and Wooller *et al*.^54^ demonstrated that presenting a realistic visual feature without sound will render an environment more fearful (participants expect something dangerous to appear) and more stressful. Similarly, Hedblom *et al*. found that adding bird songs to visual urban environments increased the positive perceptions of the urban environments^54^. We argue that adding olfactory to auditory and visual features might strengthen the overall effects due to the potential multisensory benefits. Whether effects are merely additive or are superadditive (i.e., the sum of the effects being greater than their individual parts) needs to be assessed in future studies employing a multisensory specificity design^53^. In addition, studies are needed that investigate whether the intensity of sensory stimuli influences relaxing effects. Wooller *et al*.^54^ investigating auditory, olfactory, and natural visual environments for exercise performance (not stress reduction), suggested that optimal doses of sensory stimuli may vary according to the quality of the sensory experience in specifically-simulated natural environments.

### Visual features and diversity of bird songs

Our study shows that spectacular environments (e.g., forest trails next to streams^21^ or coral reefs^40^) are not needed to promote stress reduction but rather unimpressive and common environments that might be found within urban green spaces can elicit the same stress reducing effects. That is, everyday environments can reduce stress. We used two ‘green’ environments common in cities, parks, and natural remnants of forests within the Northern hemisphere. Because urban forests are being reduced and often replaced by parks^55^, it is of interest to know their importance for well-being. We also attempted to be specific in our choice of bird species because the vocalization of birds affects attention fatigue and stress restoration differently^56^ and because we wish to avoid generalising their importance as ‘tweeting birds’ as in previous studies^15,21,35^. Moreover, we controlled for the diversity of species by including nine species in the forest environment and only one species in the park environment. Previous studies about birds have demonstrated that more species vocalizing increases the positive perception of urban environments and green spaces^42,46,57^. Therefore, we expected that increased diversity would lead to a larger differences in stress recovery between the forest and park environments. However, the importance of diversity for well-being is questioned^26^, and what constitutes diversity for the different senses is not clear. For the visual park and forest images, diversity was represented by the number of plant species, which ranged from a few to many. For the olfactory and auditory stimuli, diversity was represented by a clearer dichotomy of one versus several bird species or odour sources. Participants were not informed about this specific feature, although it is unclear whether they were aware of the diversity manipulation. Our study shows that there may be additional effects linked to senses that influence differences in diversity perception. It could also be that diversity is not linked to stress reduction, as all previous studies are based on self-evaluation. Future studies would benefit from using a parametric diversity modulation design where both the number of species and their perceived biological closeness are systematically varied to assess their individual contributions to stress reduction.

### Statistical considerations

Due to the multitude of questions we asked in this experiment, multiple testing was performed on the different sub-datasets. Increases in statistical testing increase the probability of false positives. A conservative measure is to perform a Bonferroni correction where the statistical values are corrected for the number of statistical tests performed where more tests mean that a more conservative threshold for significance is used. Because we used a large number of tests, the final statistical threshold is very conservative. Therefore, without a direct replication, these results should be considered tentative.

### Conserving nature in cities with sounds and odours for well-being

Studies of urban parks and forests are of particular interest for strategic planners and managers as presumably they want to optimise the urban dwellers’ stress-reducing environments. These areas are both visual green and allow to experience natural sounds and smells that urban environments cannot. It is important to acknowledge that if urban parks and forests are optimised to reduce stress, their overall importance to other ecosystem services, such as CO_2_ uptake, water runoff, and urban heat effects, are not diminished. On the contrary, providing areas that are optimal for stress reduction should not decrease the overall sustainability of the city because urban forests and parks improve the health and sustainability of city ecosystems. Our study showed that bird songs might play a role in multisensory stress reduction and cities provide substantial diversity of birds globally^58^. Thus, creating barriers that block noise would make it easier to hear singing birds^59^. Presently, the main focus on designing and managing public places, green or non-green, is on visual features. However, our results indicate that odour stimuli may be of high or higher importance for our well-being and urban planners should consider a city’s ‘smellscape’.

## Method

### Participants

A total of 154 individuals participated in the study using a between-group design where each participant was exposed to only one environment (each environment included a 2D 360° VR photo, sound, and smell; see experimental environments below) to reduce potential carry-over effects of the stress induction procedure. Thus, the experiment included one environment for each participant, which was assigned in a pseudo-random order based on participant entry: city (n = 50, 28 women, 22 men, mean age 27); park (n = 52, 26 women and 26 men, mean age 28); and forest (n = 52, 28 women and 24 men mean age 27). Inclusion criteria comprised self-declared health, between 18 and 50 years old, normal to corrected eye sight and hearing, normal sense of smell, self-declaration as not pregnant, and not using prescription medication. Participants’ olfactory function was assessed before the main study using a five-item, four-alternative cued odour identification test with a minimum of three or more correctly identified odours as a requirement for participation^60^. All participants demonstrated normal sensory functions. Before enrolment, all participants provided signed informed consent and all research was performed in accordance with relevant guidelines/regulations and approved by the regional ethical review board (*Etikprövningsnämnden*, Dnr: 2016/175).

### Equipment and software

The experiment included three 2D 360° Virtual Reality photos of an urban environment (densely built urban area), a forest, and an urban park. The 360 degree photos were presented using a VR mask (Oculus Rift). The photos were taken using a Ricoh Theta S camera and the software Theta A, an iOS application. Sharpness of the photos, removal of shadows created by the tripod, and light corrections were made using Photoshop. We used 2D photos because 3D photos may result in additional negative and positive interpretations (distances) and because previous studies do not use 3D photos due to these potential negative effects^40^.

*Auditory stimuli* were presented through earphones integrated in the VR mask. The bird songs played in the natural environments were mixed to simulate the natural habitat (bird song authenticities were validated by two experts). Bird songs were played in stereo, mimicking the way they are heard in nature, and songs were heard in the left and right ears at different strengths (simulating a bird either far or near). Traffic noise was played in the urban environment. The bird songs and traffic noises were downloaded from open sources on the Internet and mixed using Audacity 2.1.2. The sound levels varied depending on the ‘location’ – city: 57–79 dBA; park: 45–79 dBA; and forest: 45–78 dBA. *Olfactory stimuli* (smells from grass, fir, diesel, etc.; see experimental environments below) were presented by a computer-controlled olfactometer. Each environment had its own odour lines to avoid odour mixing. Odorants were presented birhinally with the use of a custom-built nine-channel air-dilution olfactometer^61^. Teflon tubes with an inner diameter of 1/16 inches delivered the odorized air via custom-made anatomically shaped nose pieces. Between odour presentations, a constant flow of clean air of approximately 0.3 L/m was used to clear the tubing system and the participants’ nasal cavities of odorants. The odorants were delivered at an approximate rate of 1.5 L/m or 0.75 L/m per nostril. The odours for the city environment were obtained from Unisent Scandinavia AB, the odours for the park were AAA-Aldehyde obtained from Sigma Aldrich Inc., and odours for the forest came from a variety of sources: Fir oil from *Essencefabriken*; European silver fir from Essential Oil (Pharma BA, Austria); and mushroom from Octanol (Sigma Aldrich Inc.). For the duration of the experimental session (approximately five minutes and 30 seconds for baseline, stress induction, and stress reduction), each odour was presented every five seconds for three seconds to prevent odour adaptation and nasal irritation by continuous olfactory stimulation. Between odours, the olfactory tubes were flushed with clean air for two seconds to facilitate the next presentation. Visual and auditory aspects of the environment were constantly present.

### Experimental environments

Each photo resembled common everyday environments in Sweden, specifically avoiding the spectacular visual environments used in other virtual reality studies, such as forest trails next to streams^21^ or coral reefs^40^. To avoid any distraction from the environments pictured, no people, water, or sun was seen in the photos for any environment.

Photographs for the urban environment lacked any vegetation and major commercial signs and were taken early in the morning to avoid pedestrians (Fig. 1a). To this environment, urban traffic noise (no voices) was matched and played and three city-related olfactory stimuli were presented: tar, gunpowder, and diesel. The *park environment* was dominated by lawn and managed pine trees (*Pinus sylvestris*), including some distant buildings (Fig. 1b). One bird species recording was played (Willow warbler, *Phylloscopus trochilus*), and a slight breeze was added as background noise to provide a more realistic soundscape. The olfactory stimulus presented in the park was grass. Photographs of the *forest* environment were taken in an urban forest that had not been managed for over 30 years, which resembled a primeval forest to some extent, with downed and standing dead wood, different tree layers, and mixed coniferous and deciduous trees (Fig. 1c). The forest environment included nine bird species of birds, all of which were characteristic for Swedish urban parks and forest: Willow warbler, Chaffinch (*Fringilla coelebs*), Common Black bird (*Turdus merula*), European Robin (*Erithacus rubecula*), Blue tit (*Cyanistes caeruleus*), Eurasian Nuthatch (*Sitta europaea*), Common wood pigeon (*Columba palumbus*), Great spotted woodpecker (*Dendrocopos major*), and Common swift (*Apus apus*)^42^. As in the park environment, a background breeze was added to the soundscape. The olfactory stimuli were fir, European silver fir, and mushroom.

### Stress induction and measurements

Stress was induced through minor electrical shocks generated via a PowerLab system at different intervals (Fig. 2). The shocks were delivered through electrodes on the phalanx of the index and middle fingers on the non-dominant hand. Skin conductance was measured throughout the whole experiment using Ag/AgCl electrodes on the index and middle fingers of the dominant hand. Data were acquired at a 1000 Hz rate filtered offline using a 0.1 Hz high-pass filter and a 1 Hz low-pass filter. Subjective stress sensibility was estimated using a scale of seven statements rated between 1 and 4 (1 = not correct at all and 4 = totally correct)^62^. Skin conductance was measured continuously during the whole experiment (see Fig. 2 for experimental timeline and average SCL every 20 seconds).

### Data reduction and statistical analyses

**Figure 4:**
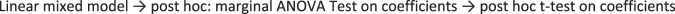
Illustrating the linear mixed model performance.

Skin conductance levels were recorded continuously for the full duration that the participants spent in the virtual environment. The first 30 seconds of the recording were used as a baseline measurement for each participant. All values in the stress induction and stress recovery periods were adjusted by the mean value of the first 30 seconds of the experiment to create a baseline-adjusted skin conductance value. For each participant, an average of the SCL was taken every 20 seconds, resulting in 15 unique data points that were then used in mixed-model analyses. Fixed effects in the linear mixed-model analyses were period (Stress and Recovery) and environment (city, park, and forest) with an interaction term, and the random effect was participant. The linear mixed model was used to reveal overall effects of period and environment, followed by a marginal post hoc ANOVA^63^ and a paired *t*-test using MATLAB 2018a Statistics and machine learning Toolbox (Fig. 4).

To assess the overall contribution of the perceived pleasantness of the environments on the SCL, regression models with step-wise entry were used. Furthermore, post hoc t-tests were used to compare differences in perceived pleasantness in the three environments.

### Experimental procedure

The experiments were conducted in a laboratory designed specifically for experimental multisensory olfactory-based testing at Karolinska Institute in Stockholm. Upon arrival, the respondents provided signed informed consent, were informed about the experiment, and were told that they would be placed in a Virtual Reality (VR) environment and be exposed to mild electrical shocks in parallel with skin conductance measurements. Because the experiment was a between group design, each participant was exposed to only one multi-sensory environment, including visual stimuli (urban area, park, and forest), olfactory stimuli (one odour or an odour mixture, if the participant was exposed to the forest environment), and auditory stimuli (at least two sounds in each environment). Participants rated the perceived pleasantness of each sensory modality from 1 (very unpleasant) to 100 (very pleasant). We used mild electrical shocks instead of stressful situations^21^ or movies^15^ to enable more equal stress responses. The intensity of the electrical shocks was determined in agreement with each respondent. An initial shock of 0.5 ampere was delivered and subsequent shocks were increased gradually by 0.3 ampere. The respondents rated the experienced shock in each increase on a 0 to 10 scale (1 = does not feel at all and 10 = hurts). When the respondent perceived a 7 (uncomfortable but not painful), the intensity was considered to be proper and that intensity was then used throughout the experiment. The participants received five electric shocks in total, one at each of the following time points: 40, 50, 70, 100, and 150 seconds. Although the participants were not aware that the stress induction period was over, they received no electrical shocks for the remaining 180 seconds (Fig. 2).

## Supplementary information

Figure SI1.
