# Supplementary material for: Reduction of physiological stress by urban green space in a multisensory virtual experiment
Source: Sci Rep. 2019 Jul 12;9:10113. doi: 10.1038/s41598-019-46099-7 (PMC6625985; doi:10.1038/s41598-019-46099-7)
Supplement: Supplementary file 1 — Figure SI1. [file 41598_2019_46099_MOESM1_ESM.docx]

Supplementary Information.

**Reduction of physiological stress by urban green space in a multisensory virtual experiment**

Marcus Hedblom^1,2*^, Bengt Gunnarsson ^3^, Behzad Iravani^4^, Igor Knez^5^, Martin Schaefer^4^, Pontus Thorsson^6^, and Johan N. Lundström^4,7,8,9^

1. Department of Forest Resource Management, Swedish University of Agricultural Sciences, Umeå. Sweden

2. Department of Ecology. Swedish University of Agricultural Sciences, Uppsala, Sweden

3. Department of Biological and Environmental Sciences, University of Gothenburg, Gothenburg, Sweden

4. Department of Clinical Neuroscience, Karolinska Institutet, Stockholm, Sweden

5. Department of Social Work and Psychology, University of Gävle, Gävle, Sweden

6. Division of Applied Acoustics, Chalmers University of Technology, Gothenburg, Sweden

7. Monell Chemical Senses Center, Philadelphia, Pennsylvania

8. Department of Psychology, University of Pennsylvania, Philadelphia

9. Stockholm University Brain Imaging Centre, Stockholm University, Stockholm, Sweden

*e-mail: [marcus.hedblom@slu.se](mailto:marcus.hedblom@slu.se)


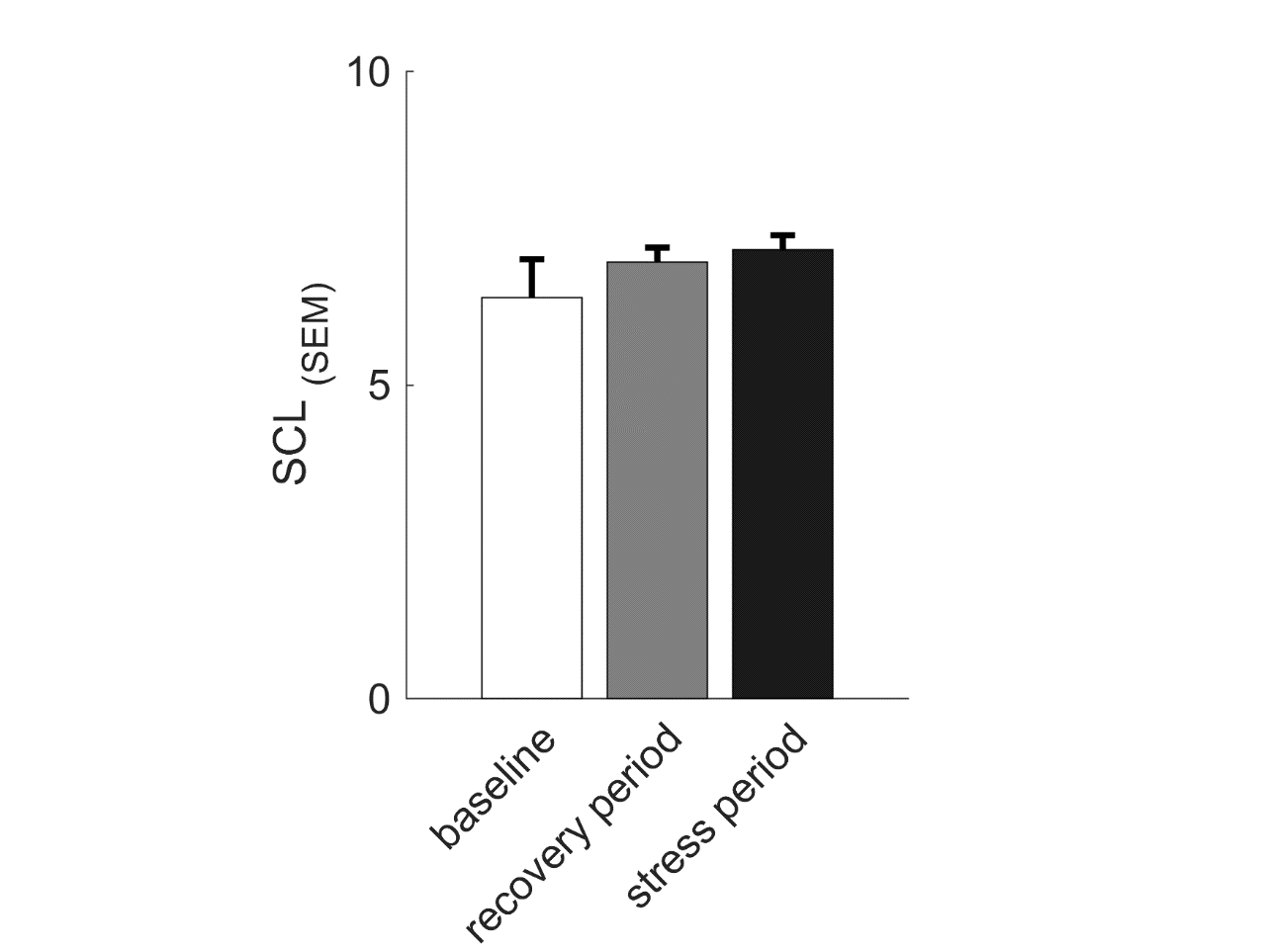


**Figure S1.** SCL increases as a function of period, suggesting successful stress induction. The figure reveals the result from post hoc analysis Marginal ANOVA test on the linear mixed effect model (SCL ~ 1 + Period + Environment + (1 | subject) + (1 | Study)).
